# Analysis of Cathepsin and Furin Proteolytic Enzymes Involved in Viral Fusion Protein Activation in Cells of the Bat Reservoir Host

**DOI:** 10.1371/journal.pone.0115736

**Published:** 2015-02-23

**Authors:** Farah El Najjar, Levi Lampe, Michelle L. Baker, Lin-Fa Wang, Rebecca Ellis Dutch

**Affiliations:** 1 Department of Molecular and Cellular Biochemistry, University of Kentucky, Lexington, Kentucky, United States of America; 2 CSIRO Australian Animal Health Laboratory, East Geelong, Victoria, Australia; 3 Program in Emerging Infectious Diseases, Duke–National University of Singapore Graduate Medical School, Singapore, Singapore; University of Missouri, UNITED STATES

## Abstract

Bats of different species play a major role in the emergence and transmission of highly pathogenic viruses including Ebola virus, SARS-like coronavirus and the henipaviruses. These viruses require proteolytic activation of surface envelope glycoproteins needed for entry, and cellular cathepsins have been shown to be involved in proteolysis of glycoproteins from these distinct virus families. Very little is currently known about the available proteases in bats. To determine whether the utilization of cathepsins by bat-borne viruses is related to the nature of proteases in their natural hosts, we examined proteolytic processing of several viral fusion proteins in cells derived from two fruit bat species, *Pteropus alecto* and *Rousettus aegyptiacus*. Our work shows that fruit bat cells have homologs of cathepsin and furin proteases capable of cleaving and activating both the cathepsin-dependent Hendra virus F and the furin-dependent parainfluenza virus 5 F proteins. Sequence analysis comparing *Pteropus alecto* furin and cathepsin L to proteases from other mammalian species showed a high degree of conservation; however significant amino acid variation occurs at the C-terminus of *Pteropus alecto* furin. Further analysis of furin-like proteases from fruit bats revealed that these proteases are catalytically active and resemble other mammalian furins in their response to a potent furin inhibitor. However, kinetic analysis suggests that differences may exist in the cellular localization of furin between different species. Collectively, these results indicate that the unusual role of cathepsin proteases in the life cycle of bat-borne viruses is not due to the lack of active furin-like proteases in these natural reservoir species; however, differences may exist between furin proteases present in fruit bats compared to furins in other mammalian species, and these differences may impact protease usage for viral glycoprotein processing.

## Introduction

In the past twenty years, bats of different species have been recognized as important hosts of viruses from different families including rhabdoviruses [1–3], coronaviruses [4–9], filoviruses [10–12], flaviviruses [13,14], orthomyxoviruses [15–17], paramyxoviruses [18,19] and others [20,21]. Numerous studies have shown that bats not only harbor a large number of viruses, but are also a major source for the emergence and transmission of viruses that cause highly pathogenic infectious diseases in humans, most importantly Severe Acute Respiratory Syndrome-like coronavirus (SARS-like CoV) [7], Ebola virus [10,22] and the henipaviruses, Hendra virus [23–26] and Nipah virus [27–29], which are members of the paramyxovirus family. Hendra virus first emerged in 1994 in Australia in an outbreak that occurred in horses [30], and more than thirty subsequent outbreaks have occurred, with a total of four human deaths associated with the virus infection [31,32]. Another closely related virus, Nipah virus was identified in Malaysia in 1999 causing an outbreak of viral encephalitis [33]; with additional outbreaks showing high mortality rates that reached 70%. Several species of bats within the genus *Pteropus*, commonly known as flying foxes, have been confirmed as the natural primary reservoir of henipaviruses [23,25,27,34–36]. Cedar virus, a novel henipavirus that does not seem to cause clinical disease in several animals which are known to be susceptible to Hendra and Nipah viruses, was identified recently and also has *Pteropus* bats as its natural reservoir [37]. Recent evidence suggests that henipaviruses are also present in non-*Pteropus* fruit bats in Africa [38,39]. Despite the important role of bats in the emergence of henipaviruses and other highly pathogenic viruses, very little is known about the viral life cycle or virus-host interactions in this natural reservoir.

Entry of henipaviruses into host cells requires fusion of the viral envelope with the cell membrane. The fusion event is mediated by two glycoproteins present on the viral envelope, the attachment protein, G, required for initial binding of the virus, and the fusion protein, F, which drives subsequent fusion of the two membranes by undergoing a series of conformational changes [40–42]. The fusion protein of paramyxoviruses is synthesized as an inactive precursor F_0_ that is cleaved by host proteases into the fusogenically active disulfide-linked heterodimer F_1_+F_2_. For the majority of paramyxoviruses, including measles virus [43], parainfluenza virus 5 (PIV5) [44] and Newcastle disease virus [45], this cleavage is mediated by the protease furin in the medial- and trans-golgi network (TGN). For some paramyxoviruses, an extracellular protease is responsible for the proteolytic activation (reviewed in [46]). However, henipaviruses are unique in that they utilize the endosomal/lysosomal protease cathepsin L, and in some cases cathepsin B, to cleave and activate the fusion protein [47,48]. This unusual role of cathepsins in the henipavirus life cycle requires a complex trafficking pathway for the activation of F protein in which the protein is synthesized and traffics to the plasma membrane in the uncleaved precursor form, F_0_. The protein is then endocytosed, cleaved in the endosomal compartments by cathepsin L or B and recycled back to the plasma membrane as the fusogenically active F_1_+F_2_ heterodimer [47–54]. The reason for this complex method of proteolytic activation remains unclear, but the cathepsin activation of henipavirus F proteins cannot be functionally replaced by other proteases, as a Nipah F protein mutant containing trypsin- or furin- cleavable sites displays reduced F processing [55]. Cleavage of the Hendra and Nipah F proteins occurs at a monobasic cleavage site GDV-K/R [56,57]; however, mutagenesis studies demonstrated that mutation of the basic residue at the cleavage site or of amino acids upstream of this site did not eliminate F protein processing [57,58], contradictory to other viral fusion proteins [59–62].

Cathepsins have been shown to be involved in the processing of several viral proteins. Cathepsin L proteolysis of the spike protein S of SARS-CoV is necessary for membrane fusion activation [63]; in addition, Ebola virus utilizes cathepsin L and B for processing and priming of the GP glycoprotein [64,65]. Interestingly, bats have been recently confirmed as the primary reservoir for SARS-CoV [8], SARS-like CoV [7] and the filovirus Marburg virus [11], while serological evidence suggests that *Rousettus aegyptiacus* fruit bats are potential reservoirs for Ebola virus [66]. This raises the question of whether the unique utilization of cathepsins by henipaviruses may be an evolutionary adaptation to the nature of proteases present in their natural reservoirs, the fruit bats. To address this, we examined the proteolytic processing of the cathepsin-dependant Hendra virus F protein and the furin-dependent PIV5 F in cells of two species of fruit bats, *Pteropus alecto* and *R*. *aegyptiacus*. Our results show that cell lines from fruit bats have both active cathepsin and furin-like proteases capable of cleaving and activating viral fusion proteins. In addition, we demonstrate that the dependence of Hendra virus on cathepsin L and vesicular trafficking for proteolytic processing of its fusion protein also occurs in cells of its natural fruit bat reservoir. Comparison of amino acid sequences of *P*. *alecto* cathepsin L and furin proteases to those of different mammalian species revealed that both cathepsin L and furin show a high degree of conservation among mammals but there are bat-specific amino acid changes, primarily in the C-terminus of *P*. *alecto* furin. Closer examination of furin-like proteases revealed that fruit bats have active furins that resemble other mammalian furins in terms of activity and response to protease inhibitors, but our results suggest differences in intracellular localization of furin in fruit bats which may influence accessibility of viral proteins to furin proteases in these natural reservoir hosts.

## Materials and Methods

### Cell lines and reagents

Vero cells, baby hamster kidney (BHK) cells and *P*. *alecto* bat cells derived from different organs, Kidney (PaKi), brain (PaBr), lung (PaLu) and fetus (PaFe) [67] were grown in Dulbecco’s modified Eagle’s medium (DMEM; Gibco Invitrogen) supplemented with 10% fetal bovine serum (FBS) and 1% penicillin and streptomycin. *R*. *aegyptiacus* fetus body cells (R06E) or head cells (R05T) [68] were maintained in DMEM-F12 media (Gibco Invitrogen) supplemented with 10% FBS and 500μg of gentamicin. A549 cells were grown in Roswell Park Memorial Institute medium (RPMI; Lonza) supplemented with 10% FBS and 1% penicillin and streptomycin. BEAS-2B cells, a human lung/bronchial epithelial cell line, obtained from ATCC were maintained in BEGM medium containing all the recommended supplements (Lonza). The protease inhibitor E64d was obtained from Sigma, cathepsin L inhibitor I and furin inhibitor, decanoyl-RVKR-chloromethylketone (dec-RVKR-CMK), were purchased from Calbiochem EMD Millipore. Fluorogenic furin substrate was obtained from Calbiochem EMD Millipore.

### Plasmids and antibodies

Hendra virus F and G coding sequences were subcloned into the pCAGGS mammalian expression plasmid as previously described [52]. pCAGGS vectors containing PIV5 F and HN genes were kindly provided by Robert Lamb (Howard Hughes Medical Institute, Northwestern University). Polyclonal antibodies (commercially produced by GenemedCustomPeptide Antibody Service, San Francisco, CA) to amino acid residues 526–539 or 516–529 in the cytoplasmic tails of Hendra virus F or PIV5 F, respectively, were used to immunoprecipitate the F protein [52].

### Expression of Hendra virus and PIV5 fusion proteins

Subconfluent monolayers of Vero cells and bat cells: R06E and PaKi were transfected with the expression vectors pCAGGS-Hendra F or pCAGGS-PIV5 F, encoding the Hendra virus F or PIV5 F proteins, using Lipofectamine Plus (Life Technologies) according to manufacturer’s protocol. Vero cells in 35-mm dishes were transfected with 2 μg of plasmid DNA, 6 μl of plus reagent and 4 μl of lipofectamine in 0.8 ml of Opti-MEM (Gibco Invitrogen). The transfection efficiency in bat cells in general was much lower than Vero cells, so transfections were performed in 100mm dishes to allow for sufficient protein expression. For expression of fusion proteins in bat cells, 12 μg of DNA, 18 μl of plus reagent, 12 μl of lipofectamine and 1.2 ml of Opti-MEM (Gibco Invitrogen) were combined and added to cells grown in 100-mm dishes. At 3–4 hours post-transfection, cells were washed with phosphate buffered saline (PBS) and incubated overnight at 37°C in DMEM or DMEM-F12 media supplemented with 10% FBS and antibiotics.

### Metabolic labeling and immunoprecipitation

Twenty-four hours post-transfection, cells were starved in cysteine- and methionine- deficient DMEM media for 45 minutes followed by labeling in Tran^35^S-label (100 μCi/ml; Perkin Elmer, Waltham, Massachusetts). To determine total expression of fusion proteins, cells were labeled for 3 hours at 37°C and lysed immediately. For pulse chase experiments, cells were labeled for 30 minutes, washed twice with PBS, normal DMEM or DMEM-F12 media was then added, and cells were chased for varying times. At the end of the chase periods, cells were washed and lysed in radioimmunoprecipitation assay (RIPA) lysis buffer (100 mM Tris-HCl [pH 7.4], 150 mM NaCl, 0.1% SDS, 1% Triton X-100, 1% deoxycholic acid) containing 0.15 M NaCl and supplemented with protease inhibitors. Lysates were then clarified by centrifugation at 136,000xg for 15 minutes at 4°C and supernatants were immunoprecipitated with anti-peptide sera to the F proteins and protein-A conjugated sepharose beads [69]. Immunoprecipitated proteins were analyzed on 10% sodium dodecyl sulfate-polyacrylamide gel electrophoresis (SDS-PAGE) and visualized using the Typhoon imaging system (Amersham Biosciences/GE Healthcare Life Sciences, New Jersey). ImageQuant TL (GE Healthcare, Piscataway, NJ) was used to determine band densitometry and results were expressed as percent cleavage defined as F_1_/(F_1_+F_0_).

### Syncytia assay

Vero cells or PaKi cells in 35-mm dishes were transiently transfected with Hendra virus F or PIV5 F alone or in combination with the homotypic attachment protein (G or HN). The F:G/HN ratio used was 1:3 for Hendra virus and 1:1 for PIV5. Twenty-four to 48 hours post transfection, syncytia formation was examined and photographs were taken using a Nikon digital camera mounted atop a Nikon TS100 microscope with 10x objective.

### Furin-like enzyme activity

A furin-like enzyme activity assay on whole cell lysates was performed as described [70] with minor modifications. 2×10^6^ cells were collected, washed with PBS and lysed for 10 minutes on ice in 200 μl of 5× lysis/reaction buffer (500 mM HEPES, pH 7.0, 2.5% Triton X-100, 5 mM calcium chloride, 5 mM β-mercaptoethanol). Cells were then sheared with a 23-gauge needle followed by centrifugation at 13,000xg for 10 minutes at 4°C, and supernatants were stored at -80°C. For determination of furin-like enzyme activity, cell lysates were diluted 2 fold in 5x lysis buffer. In a black opaque 96-well plate, 20 μl of cell lysates were added to 70 μl of ultrapure water and the plate was incubated for 15 minutes at 37°C. After incubation, 10 μl of 1 mM furin fluorogenic substrate, previously pre-warmed at 37°C for 30 minutes, was added and fluorescent intensity was immediately measured on a SpectraMax Gemini XPS plate reader (Molecular Devices) every 3 minutes for 240 minutes with excitation at 355 nm and emission at 460 nm. For determination of the effect of a furin inhibitor on furin-like activity, cell lysates were incubated with increasing concentrations of the inhibitor for 3 hours at 37°C and cells were then processed for the enzyme activity assay as mentioned above.

### Multiple sequence alignment of mammalian furin and cathepsin L

Sequences of *P*. *alecto* furin and cathepsin L were identified using BLAST searches of the P. alecto genome and transcriptome databases generated previously [71,72]. Sequences of other mammalian proteases were obtained from GenBank. Multiple sequence alignment of mammalian proteases was generated using ClustalW [73]. *P*. *alecto* bat furin or *P*. *alecto* cathepsin L was used as a standard reference for amino acid numbering.

## Results

### The Hendra virus and PIV5 fusion proteins are efficiently cleaved in fruit bat cells

**Fig 1:**
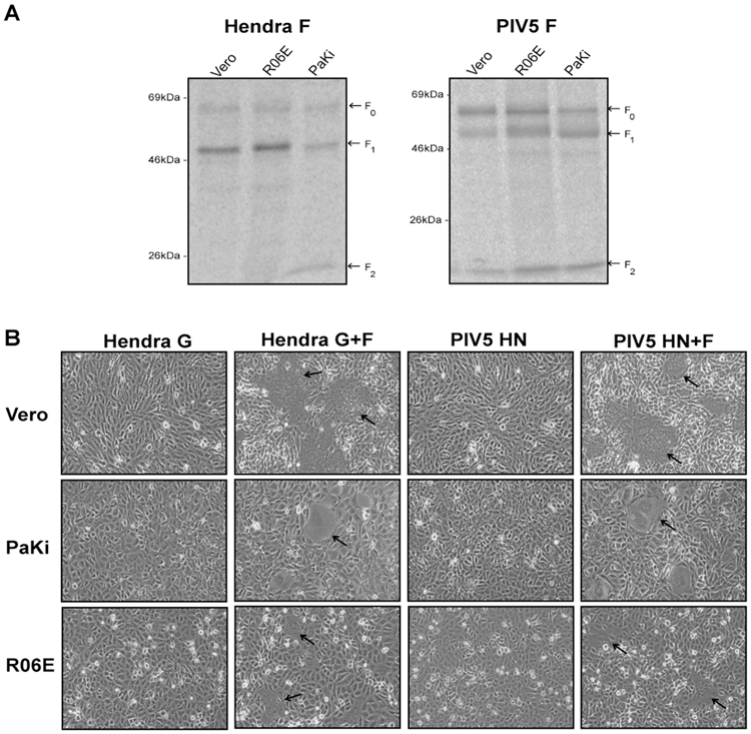
Bat cells cleave cathepsin-dependant Hendra virus F and furin-dependant PIV5 F. (A) Cells were transfected with pCAGGS-Hendra virus F or pCAGGS-PIV5 F and 18–24 hours post transfection, metabolically labeled with Tran ^35^S for 3 hours at 37°C. Following labeling, cells were lysed and immunoprecipitated. Proteins were analyzed by 10%SDS-PAGE and visualized by autoradiography. (B) Cells were transfected with Hendra virus F or PIV5 F alone or in combination with Hendra virus G or PIV5 HN. 24 to 48 hours post transfection, cells were washed and images were taken using a Nikon digital camera mounted atop a Nikon TS100 microscope with 10x objective. Arrows indicate syncytia.

Several cell lines derived from different bat species have been established, providing a valuable tool for *in vitro* studies of virus life cycles in their natural reservoir. In this study, we utilized cells previously established from two pteropid fruit bats, *P*. *alecto* [67] and *R*. *aegyptiacus* [68]. *P*. *alecto* cells derived from different tissues were shown to be permissive to henipavirus replication and cells derived *R*. *aegyptiacus* permitted filovirus infection, indicating that these cells contain the necessary host factors required for virus replication [67,74]. However, very little is currently known about the nature of proteases present in these bat species. To assess the ability of *pteropus* host cell proteases to proteolytically process viral fusion proteins, we examined the proteolytic processing of the cathepsin-dependent Hendra virus F protein and the furin-dependent PIV5 F protein in *P*. *alecto* kidney cells (PaKi) and *R*. *aegyptiacus cells* obtained from body tissues (R06E). Bat cells and Vero cells, used as a control, were transiently transfected to express the Hendra virus F or PIV5 F protein and metabolically labeled. The fusion proteins were subsequently immunoprecipitated and analyzed on 10% SDS-PAGE. As seen in Fig. 1A, both fusion proteins were proteolytically processed into the F1 and F2 heterodimer in R06E and PaKi cells. This indicates that cells from both *P*. *alecto and R*. *aegyptiacus* have active cathepsin-like and furin-like proteases capable of cleaving the Hendra viurs and PIV5 F proteins. To assess whether the processed proteins were fusogenically active, syncytia assays were performed. Syncytia formation was not observed in the presence of the attachment (Hendra G or PIV5 HN) protein alone, consistent with the role of the F protein in promoting fusion. However, syncytia were observed in all three cell types upon expression of the fusion and attachment proteins of either Hendra virus or PIV5 (Fig. 1B, arrows). Syncytia formed in R06E and PaKi were smaller in size compared to syncytia seen in Vero cells and the total number of syncytia observed in the two fruit bat cell lines was less than in Vero cells, likely as a result of lower transfection efficiency in bat cells versus Vero cells. These results indicate that *P*. *alecto and R*. *aegyptiacus* fruit bat cells can cleave and activate cathepsin-dependent and furin-dependent viral fusion proteins.

### Kinetics of PIV5 F processing differs between Vero cells and fruit bat cells

**Fig 2:**
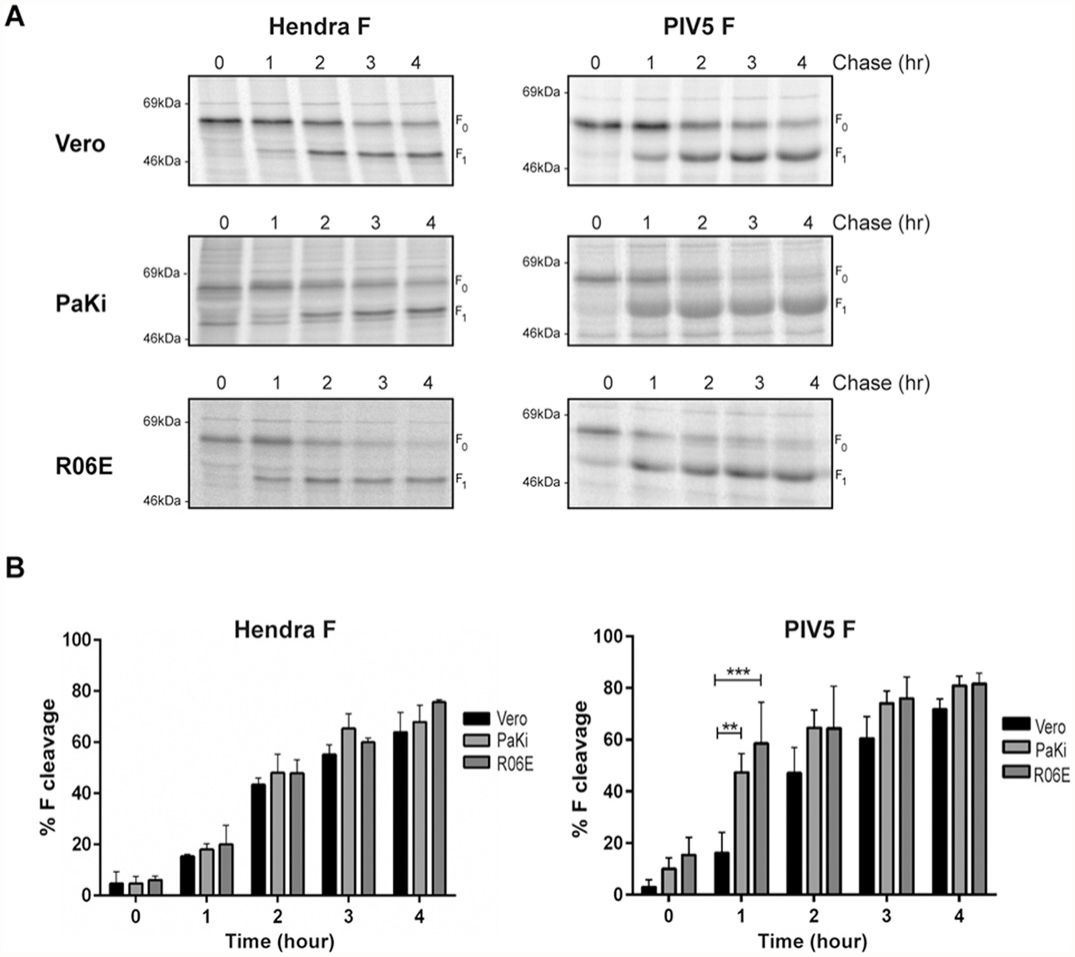
Kinetics of PIV5 Fusion protein cleavage is faster in bat cells compared to Vero cells. (A) Cells transiently transfected with pCAGGS-Hendra virus F or pCAGGS-PIV5 F were metabolically labeled with Tran ^35^S for 30 minutes and chased for the indicated times. Cells were immediately lysed and cell lysates were immunoprecipitated. Proteins were migrated on 10% SDS-PAGE and analyzed by autoradiography. (B) Quantification of F1 densitometry was done using ImageQuant, TL software (GE Healthcare, Piscataway, NJ) and results are represented as percent cleavage defined as F_1_/(F_1_+F_0_). Error bars represent the mean ± standard deviation for three independent experiments. Two-way ANOVA, **p<0.01, ***p<0.001.

The Hendra virus fusion protein undergoes a complex trafficking pathway for cleavage and activation [47,51,52], while PIV5 F is cleaved by furin as it passes through the TGN [44]. It has been previously shown that the majority of Hendra virus F cleavage occurs within 4 hours of protein synthesis [52]. To compare the kinetics of processing of Hendra virus and PIV5 F proteins in bat cells versus Vero cells, cleavage was monitored by pulse chase analysis. Vero cells or fruit bat cells were labeled for 30 minutes, washed and incubated for 0 to 4 hours in chase media. Directly following labeling (0 hours), almost only the uncleaved F_0_ form of Hendra virus F was detected in all cell types, while a small percentage of PIV5 F_0_ was cleaved to F_1_ in bat PaKi and R06E cells (Fig. 2A). Cleavage of F_0_ to F_1_ increased during the 4 hour chase period in all cell types for both Hendra virus F and PIV5 F. For Hendra virus F, R06E and PaKi cells showed similar processing kinetics to Vero cells, with no significant difference in percentage of cleavage observed at any point following synthesis. In contrast, PIV5 F was cleaved more rapidly in both bat cell types compared to Vero cells. After one hour of synthesis, more than 40% of PIV5 F_0_ had been proteolytically processed in both PaKi and R06E fruit bat cell lines, a significantly higher level than the 17% cleavage observed in Vero cells (Fig. 2B). As similar processing kinetics were observed for Hendra virus F protein, it is unlikely that the differences observed for PIV5 F are due to changes in the rate intracellular of trafficking in fruit bat cells. Cleavage of PIV5 F by furin generally occurs in secretory vesicles budding from the TGN. Thus, these data suggest a potential difference either in the intracellular localization or expression of furin present in PaKi and R06E cells compared to Vero cells.

### Hendra virus F cleavage in fruit bat cells depends on vesicular trafficking and cathepsin L

**Fig 3:**
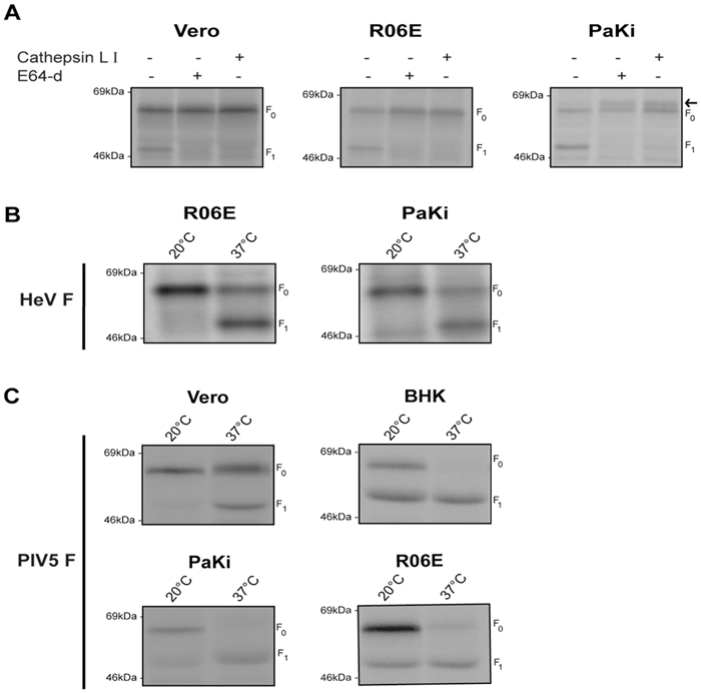
Inhibition of cathepsin L and vesicular trafficking prevent cleavage of Hendra virus F. (A) Vero cells or bat cells were transfected with pCAGGS-Hendra virus F and 24 hours post transfection, cells were metabolically labeled with Tran ^35^S in the absence or presence of the indicated inhibitor. E64-d and cathepsin L inhibitor I were added at 20μM. Cells were lysed, immunoprecipitated and anaylzed on 10% SDS-PAGE. Arrow indicates the position of a novel band. (B, C) Cells transiently transfected with Hendra virus F (B) or PIV5 F (C) were labeled with Tran ^35^S for 45 minutes and then chased for 3 hours at either 20°C or 37°C. Following lysis and immunoprecipitation, proteins were run on 10% SDS-PAGE and visualized by autoradiography.

The requirements for the activation of henipavirus fusion proteins differ remarkably from other paramyxovirus F proteins. Cleavage of Hendra and Nipah F proteins occurs by the action of cathepsin L at a monobasic cleavage site GDV-K/R [56,57]. While the kinetics of processing in bat cells were consistent with cleavage following trafficking to the endosome (Fig. 2), the dependence of henipaviruses on the endosomal cysteine protease cathepsin L in their natural reservoir was verified using non-specific and specific cathepsin inhibitors. Treatment of PaKi and R06E bat cells with the general cysteine protease inhibitor E-64d, which inhibits calpain and cathepsins B, H and L [75] prevented cleavage of Hendra virus F in all cell types (Fig. 3A). To verify that cathepsin L is specifically involved in Hendra F proteolytic processing in bat cells, an inhibitor that targets cathepsin L was used. Similar to Vero cells, inhibition of cathepsin L also ablated cleavage of F_0_ into the F_1_ and F_2_ heterodimer, indicating that processing of Hendra virus F protein is under the control of cathepsin L in its natural reservoir host (Fig. 3A). Interestingly, in PaKi cells, an extra band of higher molecular weight, which is not detected in other cell lines, was seen above F_0_ upon inhibition of Hendra F processing. A similar band was seen when a cell line derived from *P*. *alecto* brain (PaBr) was treated with E64-d and cathepsin L inhibitor (data not shown). This suggests that additional post translational modifications may occur in the uncleaved Hendra F protein in its natural reservoir *P*. *alecto*.

Proteolytic activation of Henipavirus F protein requires endocytosis [49–52], cleavage by cathepsin L and recycling of the cleaved F_1_-F_2_ heterodimer to the cell surface [47,48]. Temperature block experiments have been used to influence both exocytic and endocytic transport [76,77]. We therefore determined the effect of reduced temperature on the cleavage of Hendra virus F and PIV5 F. Cells expressing Hendra virus F or PIV5 F were metabolically labeled and chased for 3 hours either at 20°C or 37°C. The cleaved F_1_ product of Hendra F and PIV5 F was observed following incubation at 37°C. While a background band of slightly lower molecular weight than Hendra F_1_ was seen, incubation of R06E and PaKi at 20°C abolished proteolytic processing of Hendra F in bat cells (Fig. 3B), as was previously shown in Vero cells [52], consistent with the cleavage of Hendra F depending on temperature-sensitive vesicular trafficking in bat cells. Interestingly, while incubation of Vero cells at 20°C abolished PIV5 F processing, the F_1_ cleavage product could still be observed in PaKi and to an even greater extent in R06E cells (Fig. 3C). To determine whether this was specific for bat cells, we utilized an additional mammalian cell line from baby hamster kidney cells, BHK. Similar to fruit bat cells, incubation of BHK cells at 20°C did not completely inhibit PIV5 F proteolytic processing. In addition, while the majority of PIV5 F_0_ was cleaved to F_1_ in BHK, PaKi and R06E cells upon incubation for 3 hours at 37°C, F_0_ was clearly visible in Vero cells at this time point. Furin is primarily located in the Golgi and TGN, and it can also circulate between the cell surface and the TGN [78–80]. Inhibition of Hendra virus F cleavage by lowering the temperature to 20°C (Fig. 3B) indicate that endosomal trafficking is blocked in R06E and PaKi cells under this condition; however the varying effects of lower temperature on PIV5 F processing in different cell types suggest that the temperature dependence of trafficking through the TGN may differ between different cell types. These results, combined with our previous findings on the more rapid furin processing of PIV5 F in bat cells, suggest that subtle differences in cellular distribution and localization of furin or trafficking through TGN may exist between different mammalian species.

### Effect of dec- RVKR-cmk on furin-like proteases in fruit bat cells

**Fig 4:**
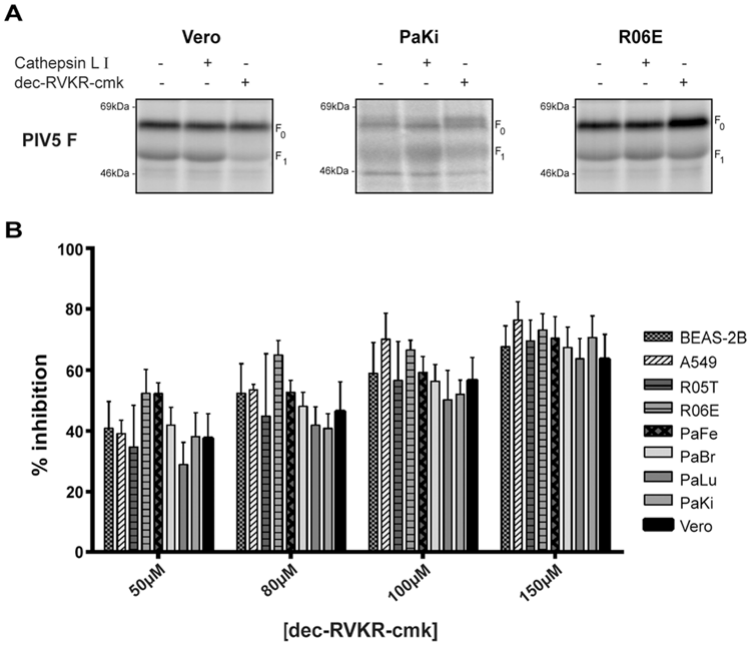
Effect of dec-RVKR-cmk on PIV5 F cleavage and furin enzyme activity in fruit bat cells. (A) Cells transfected with pCAGGS-PIV5 F were labeled with Tran ^35^S for 3 hours in the absence or presence of cathepsinL I (20μM) or dec-RVKR-cmk (50 μM). Prior to SDS-PAGE analysis, cells were lysed and subject to immunoprecipitation. Images were visualized by autoradiography. (B) 2×10^6^ cells of each cell type were lysed for 10 minutes on ice followed by shearing with a 10-gauge needle. Cell lysates were incubated with increasing concentrations of dec-RVKR-cmk for 3 hours at 37°C prior to addition of Pyr-Arg-Thr-Lys-Arg-AMC furin substrate. End-point fluorescence was measured after 4 hours using an XPS plate reader. Error bars represent the mean ± standard deviation for three independent experiments.

Results in Fig. 1A demonstrated that bat cells can cleave the fusion protein of PIV5, which is proteolytically processed by furin in other cell types [44], suggesting that bat cells have active furin or furin-like proteases. To verify the involvement of a furin protease in the cleavage of the PIV5 fusion protein, we utilized a small molecule inhibitor of furin, decanoyl-Arg-Val-Lys-Arg-chloromethyl ketone (dec-RVKR-cmk), which binds the catalytic sites of all seven mammalian proprotein convertases and inhibits their activity [81,82]. This inhibitor has also been shown to be effective in inhibiting Kex-2, the yeast endoproteinase homologue of furin as well [83]. Vero cells and fruit bat cells were treated with inhibitors of furin and cathepsin L, and cleavage of PIV5 fusion protein was assessed. Addition of cathepsin L inhibitor to Vero, PaKi or R06E cells had no effect on proteolytic processing of PIV5 F protein (Fig. 4A). In Vero cells, addition of the potent furin inhibitor dec-RVKR-cmk resulted in a marked decrease in cleavage of PIV5 F_0_ to F_1_. In contrast, the effect of the inhibitor on processing of PIV5 F in PaKi and R06E cells was minimal (Fig. 4A). Dec-RVKR-cmk has been widely used to prevent the proteolytic activation of a variety of viral glycoproteins [84–87], and inhibition of cleavage usually requires a concentration range from 25 μM to over 40 μM. In the presence of 50 μM dec-RVKR-cmk, the percentage of inhibition of PIV5 F cleavage in Vero cells was approximately 70% compared to the control without inhibitor, while only 20% inhibition in PaKi and RO6E cells was observed compared to the control. We next determined the effect of the inhibitor on furin-like enzyme activity in cell lysates from different fruit bat cells and other mammalian cells, Vero cells, A549, and BEAS-2B. Cell lysates prepared from equal number of cells for each cell type were incubated with increasing concentrations of dec-RVKR-cmk (50 μM, 80 μM, 100 μM,150 μM) for 3 hours at 37°C prior to the addition of the flourogenic furin substrate, Pyr-Arg-Thr-Lys-Arg-AMC, and release of the fluorescent AMC product was subsequently determined. All cell types showed a dose-dependent response to the drug, and as shown in Fig. 4B, there were no significant statistical differences in the effect of the inhibitor between the different cell types at all the tested concentrations, indicating that binding of the competitive inhibitor dec-RVKR-cmk to the catalytic site of furin-like proteases is comparable in the different cell types. The observed differences in the response to the potent furin inhibitor in fruit bat cells seen in Fig. 3A may therefore reflect differences in the accessibility of the inhibitor to the enzyme, i.e. the level of uptake and metabolism between the different cell types, or differences in the expression or localization of furin-like proteases.

### Bat lung cells display slower kinetics of furin-like enzyme activity than human lung cells

**Fig 5:**
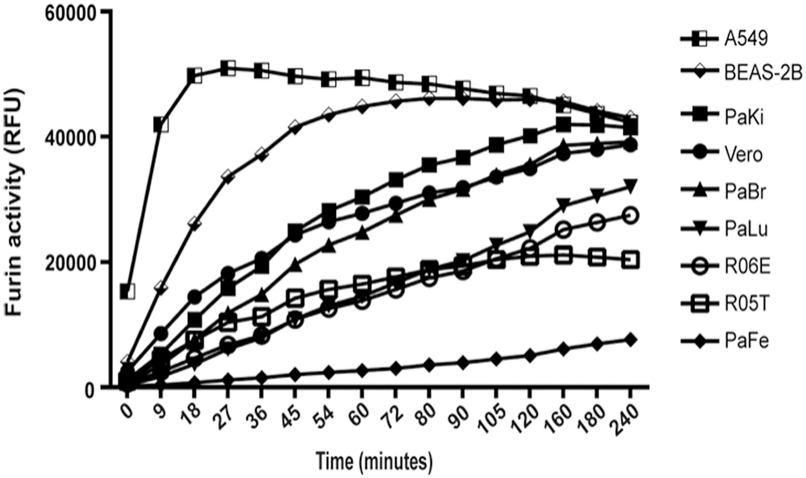
Different mammalian cell types show differences in furin-like enzyme activity. 2×10^6^ cells of each cell type were lysed for 10 minutes on ice followed by shearing with a 10-gauge needle. Clarified lysates were then incubated with 10 μM of Pyr-Arg-Thr-Lys-Arg-AMC furin substrate for 4 hours at 37°C with fluorescence measured every 3 minutes. Each cell type was assayed in duplicate and the progress curves are representative of 3 separate experiments.

Fruit bat cells have functional furin-like proteases, and processing of PIV5 F occurs more rapidly in these cells than in Vero cells (Fig. 2A). To compare the enzymatic activity of furin homologs present in bat cells to that present in other mammalian cell types, we performed a furin-like enzyme activity assay. This assay, which is not specific for furin but determines the activity of all proprotein convertases, allows determination of endogenous enzymatic activity of furin-like enzymes in whole cell lysates [70], and was used to compare the kinetics of the protease activities per cell in different cell lines. Cell lysates from 2×10^6^ cells of each cell type were added to the flourogenic substrate, Pyr-Arg-Thr-Lys-Arg-AMC, and fluorescence was monitored for 4 hours. The progress curves obtained for each cell type allowed determination of differences in total furin-like enzyme activity (Fig. 5). *P*. *alecto* fetus cells (PaFe) generated the lowest total amount of fluorescent product at each time point, indicating that both the rate and extent of furin processing per cell is lowest in these cells. *R*. *aegyptiacus* cells R06E and R05T displayed the next lowest furin-like activity. Statistical analysis showed that there was no significant difference in furin-like activity between Vero and PaKi cells suggesting that the total cellular furin-like activity is comparable in these two kidney cell lines. However, comparison of AMC release in R06E to Vero or PaKi cells showed significant difference starting 18 minutes after addition of the substrate (p value <0.05) and during the 4 hour incubation period, with a p value <0.0001 between 30 and 90 minutes. Furin-like activity in cells obtained from *P*. *alecto* brain (PaBr) was significantly higher than the other fruit bat cells *P*. *alecto* lung (PaLu) and PaFe, R06E and R05T one hour post incubation with the substrate. Interestingly, the progress curves for the total furin-like activity in the two lung human cell lines, A549 and BEAS-2B, were different from other tested cell lines. Release of AMC was faster and fluorescence reached maximum levels by 30 minutes or 60 minutes in A549 and BEAS-2B, respectively. In contrast, this rapid proteolysis of the Pyr-Arg-Thr-Lys-Arg-AMC substrate was not observed in PaLu cells and the total furin-like activity in the *P*. *alecto* lung cells was significantly lower than that of A549 and BEAS-2B cells at all timepoints, with a p value <0.0001 during the first three hours. These data reveal that bat cells have functional and active furin-like enzymes that can recognize and cleave a furin substrate, with variation seen in the total furin-like enzyme activity between cells derived from different tissues. Furin-like pro-protein convertases are expressed differently in various body tissues and the variation seen in the total furin-like activity between the different cells types is expected. However, the significant difference between the processing of the furin substrate between *P*. *alecto* lung cells and the two human lung cells suggest differences either in the activity or in the expression levels of the furin-like proteases in the lungs of the two species.

### Bat furin and cathepsin L proteases have specific amino acid sequence variations not detected in other mammalian counterparts

**Fig 6:**
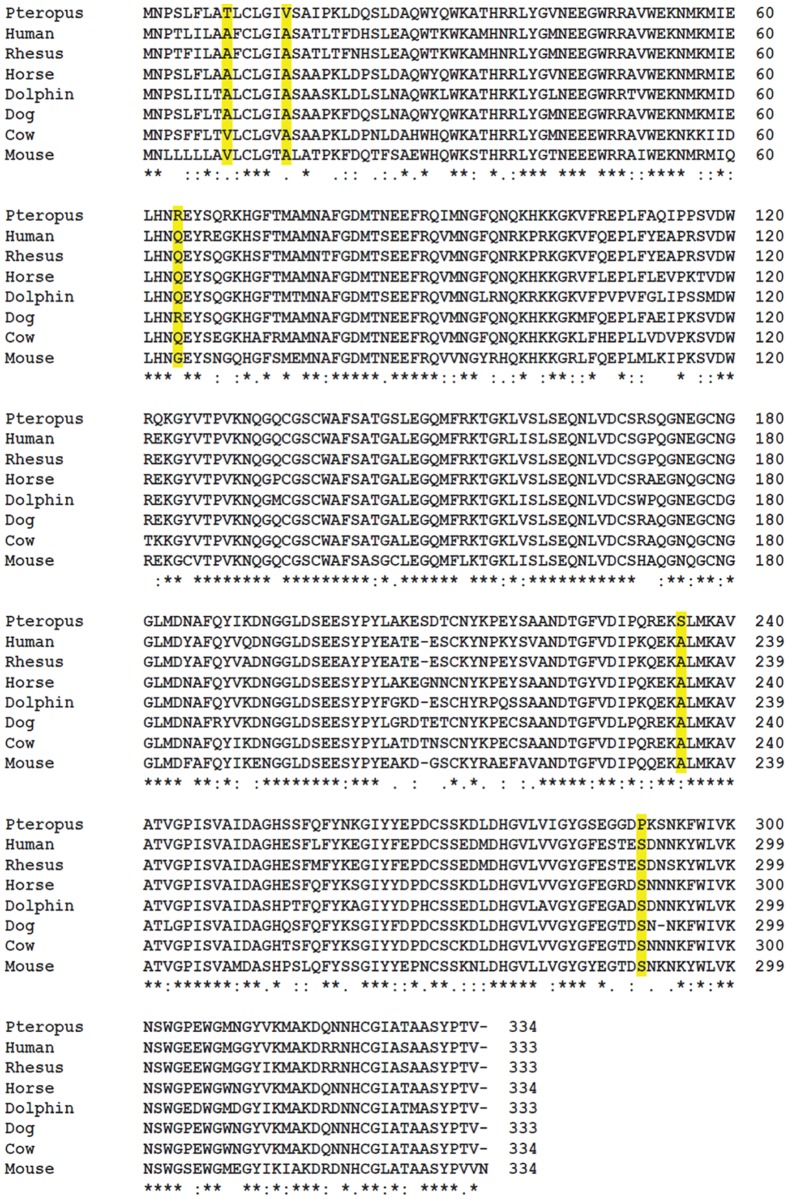
Sequence alignment of *Pteropus alecto* cathepsin L1 and cathepsin L1 of other mammalian species show Pteropus-specific amino acid changes. Sequence alignment was generated using ClustalW. (GenBank accession numbers are given in parentheses): human (P07711.2), rhesus macaque (EHH24212.1), horse (XP_001494409.1), dolphin (XP_004320974.1), dog (Q9GL24.1), cow (P25975.3), mouse (NP_034114.1). Yellow indicates amino acid changes that are specific to *P*. *alecto* cathepsin L. The asterisk “*” indicates identical residues, “:” indicates conserved substitutions and “.” semi-conserved substitutions.

**Fig 7:**
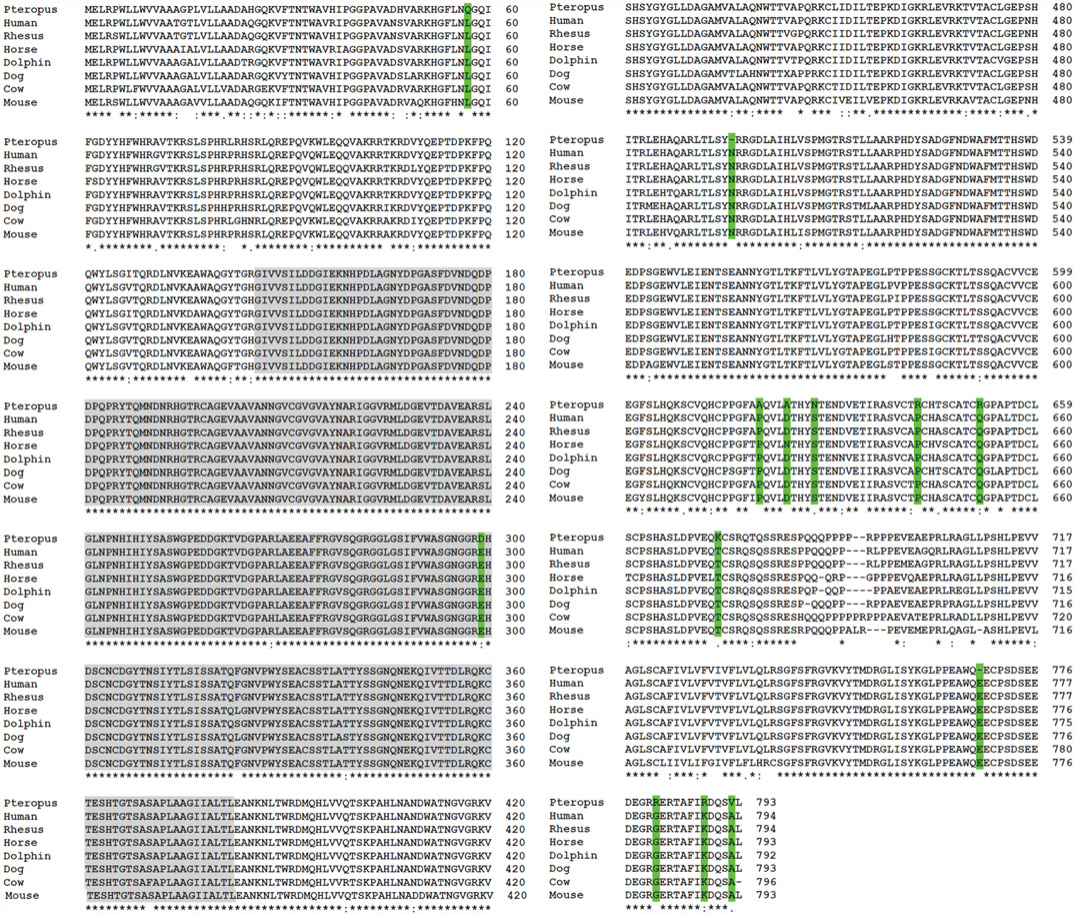
The C-terminus region of *P*. *alecto* furin has the highest degree of amino acid variation compared to furin proteases from other mammalian species. Sequence alignment was generated using ClustalW. (GenBank accession numbers are given in parentheses): human (NP_002560.1), rhesus macaque (EHH27600.1), horse (XP_005602832.1), dolphin (XP_004322334.1), dog (XP_850069.2), cow (NP_776561.1), mouse (NP_001074923.1). Highlighted in gray is the catalytic site of furin and in green the changes in amino acid residues in *P*. *alecto* compared to other mammalian furins. The asterisk “*” indicates identical residues, “:” indicates conserved substitutions and “.” semi-conserved substitutions.

To compare amino acid sequences of bat cathepsin L and furin to other mammalian proteases, we performed multiple sequence alignment analysis. Whole genome sequencing of different bat species has been performed [72,88]; however the furin sequences from both *P*. *vampyrus* and *Myotis davidii* were not complete. Sequences of furin and cathepsin L from the *P*. *alecto* transcriptome were previously generated [71]. Multiple sequence alignments of furin and cathepsin L1 amino acid sequences from *P*. *alecto* and a variety of other mammals were performed using ClustalW [89]. Both furin and cathepsin L1 showed a high level of conservation among different mammalian species however, furin showed a higher degree of conservation. The sequence alignment for mammalian cathepsin L1 proteases (Fig. 6) shows amino acid changes between the different species spread across the whole protein sequence, with some amino acid changes that are specific to *P*. *alecto* cathepsin L (marked in yellow). Compared to cathepsin L1, furin from various mammalian species had fewer amino acid changes across the entire sequence (Fig. 7). Furin is a type I membrane protein composed of an N-terminal pro-peptide followed by a catalytic site, a P/homo B domain which is essential for activity of the catalytic domain, and a C-terminal region containing a transmembrane domain [90]. The N-terminal region of *P*. *alecto* furin contained one amino acid change not observed in any other mammalian furin, as the leucine at position 57 in other mammalian furins is substituted by glutamine in *P*. *alecto*. The catalytic site (shown in gray), encompassing amino acid residues G146 to L382 [45,90] was extremely conserved, with a single glutamate to aspartate change in the catalytic site of *Pteropus* furin at position 299. The highest degree of variation observed for *P*. *alecto* furin occurred at the C-terminus, including the following unique amino acid changes: A at position 619 in *P*. *Alecto* furin in contrast to P at this position in all other mammalian furins (P619>A), D623>A, S627>N, P642>R, Q651>R, T673>K, G781>R, K788>R, A792>V, deletion of N at position 496 and deletion of E at position 768 (highlighted in green). The cytoplasmic tail controls the trafficking and cellular localization of furin. The sequences at the cytoplasmic tail of furin that are known to be critical for intracellular trafficking of furin include the acidic cluster (EECPpSDpSEEDE) and the two membrane proximal motifs YKGL and LI [78,79,91–93]. The YKGL and LI motifs are conserved in *P*. *alecto*; however, the first aspartate (position 768) in the acidic cluster sequence, which is required for phosphorylation by casein kinase II, is absent in *P*. *alecto* furin. It is possible that the deletion of the acidic aspartate affects the phosphorylation of serine 772 and thus alters the intracellular localization or distribution of furin in *P*. *alecto*.

## Discussion

Bats have recently been shown to carry a number of novel viruses [94]; however, our knowledge of the natural history of viruses in their bat reservoir host and the special features of bats that allow them to co-exist with this wide range of viruses is limited. Bats and bat-derived cells are susceptible to infection by many viruses including filoviruses, paramyxoviruses, coronaviruses and influenza virus [74,95] indicating that bats have the necessary cellular factors to mediate many viral infections. Cellular proteases play an essential role in proteolytic activation of the majority of viral glycoproteins and in the spread of infection, but very little is currently known about the protease profile of the bat hosts. Interestingly, a number of bat-borne viruses utilize the endosomal cathepsin proteases during their life cycle [47,48,63–65], in contrast to the more common use of furin proteases for intracellular viral glycoprotein processing. To address the ability of bat cells to proteolytically process viral fusion proteins, we examined the proteolytic processing of the PIV5 F protein, normally cleaved by furin, and the Hendra virus F protein, normally cleaved by cathepsin L, in cells derived from two species of bats of the Pteropodidae family. We showed that *P*. *alecto* and *R*. *aegyptiacus* have homologues of cathepsin and furin proteases capable of cleaving and activating cathepsin-dependent (Hendra virus F) and furin-dependent (PIV5 F) viral fusion proteins. This finding is consistent with previous studies showing that cells from different bat species can cleave glycoproteins of some viruses such as Ebola virus [96], and an African henipavirus [97,98]. Our data also indicate that the requirements for proteolytic processing of Hendra virus F in bat cells are analogous to those previously determined in Vero cells [47,52]. Temperature reduction experiments or inhibition of cathepsin L prevented both cleavage of Hendra virus F and syncytia formation (data not shown), indicating that vesicular trafficking and a bat homolog of cathepsin L are involved in activation of Hendra virus F in bat cells. In addition, we did not detect a significant difference in the kinetics of Hendra F cleavage in PaKi or R06E compared to Vero cells and levels of cleaved F_1_ on the cell surface of Vero cells and bat cells were similar (data not shown). These results indicate that Hendra virus F trafficking in bat cells is analogous to that in Vero cells, suggesting that Hendra virus evolved its dependence on cathepsin L to mediate infection through adaptation in its bat natural host.

Cleavage of PIV5 F protein and the furin-like enzyme activity assay indicate that bat cells from *P*. *alecto* and *R*. *aegyptiacus* have active furin-like proteases capable of recognizing and cleaving the furin consensus site R-X-K/R-R in PIV5 F protein and in the fluorogenic furin substrate. These results support recent evidence that R06E cells and other cells derived from different *Pteropodidae* bat species are sensitive to infection of viruses that utilize furin for mediating infection including filoviruses and paramyxoviruses [74,95,96]. However, kinetics of PIV5 F cleavage indicated that proteolytic processing of the furin-dependent PIV5 F is more rapid in bat cells than in Vero cells with the most rapid cleavage seen in R06E cells; however, the total furin-like enzyme activity assay showed lower total cellular furin-like activity in R06E cells compared to Vero cells. A more rapid cleavage of PIV5 F even with less furin activity per cell could result from differences in the cellular localization of the furin homologues in the bat cell types. Consistent with this, we found that reduction of temperature to 20°C did not completely inhibit cleavage of PIV5 F in R06E cells and PaKi cells, but significantly reduced proteolysis of PIV5 F in Vero cells as was previously shown [52]. Furin is a membrane-bound protease that circulates between the cell surface and the TGN through endosomes [79]; however, the cellular localization and distribution of furins may vary between the different cell types. Specific motifs in the cytoplasmic tail at the C-terminus of furin control its intracellular trafficking [78,91]. Amino acid sequence alignment of *P*. *alecto* furin and multiple other mammalian furins shows that the two membrane proximal motifs YKGL and LI required for trafficking of furin from the TGN to endosomes and the CPpSDpSEEDE motif that is important for retention of furin in the TGN are conserved in *P*. *alecto* furin [91,99]. The phosphorylated acidic cluster (EECPpSDpSEEDE) which directs trafficking from endosomes to the TGN [92] lacks the first glutamate in furin from *P*. *alecto*. In addition, several differences in specific amino acid residues occur at the C-terminus of *P*. *alecto* furin compared to other mammalian furins that may influence the localization of furin in *P*. *alecto* cells and possibly other members within the pteropodidae family.

Our data also show differences between the total cellular activity of furin-like enzymes in various cell types. Furin is ubiquitously expressed at different levels in all tissues [100,101]. The mRNA levels of furin determined in different tissues of an African monkey showed highest levels in kidney and liver, lower levels in brain, spleen and thymus and lowest levels were detected in tissues from lung, heart and testis [101]. Our results show that *P*. *alecto* kidney cells had higher activity than other *Pteropus* cell types, followed by brain cells (PaBr), lung cells (PaLu) and fetus cells, which showed the lowest furin-like activity. Interestingly, furin-like activity in PaLu cells was significantly lower and showed slower kinetics relative to the two human lung cell lines, A549 and BEAS-2B. This finding could indicate either that human lung cells have a greater number of active furin-like enzymes than *P*. *alecto* lung cells, or that the individual furin proteases in human lung cells have increased proteolytic activity. BEAS-2B cells are isolated from human bronchial epithelium, A549 are type II alveolar basal epithelial cells while the PaLu cells are mainly cuboidal epithelial cells derived from lung tissues [67]. Differences in cell type between these three cell lines may also contribute to the differences seen in the total furin-like activity.

In conclusion, our results show that bats have cathepsin-like and furin-like proteases analogous to their counterparts in other mammalian species, suggesting that the utilization of cathepsins for viral glycoprotein processing in a number of bat-resident viruses is not due to a lack of furin-like enzymes in the bat reservoir host. However, potential alterations in furin localization or activity in the bat host may affect virus replication. Newly emerging viruses can be major threats to public health, so further investigation of virus biology in bat reservoirs is needed to provide a global perspective on the changes that occur in viruses within their natural hosts that contribute to the emergence of the virus and transmission to other species.

## References

[1] Iwasaki T, Inoue S, Tanaka K, Sato Y, Morikawa S, et al (2004) Characterization of Oita virus 296/1972 of Rhabdoviridae isolated from a horseshoe bat bearing characteristics of both lyssavirus and vesiculovirus. Arch Virol 149: 1139–1154. 15168201 10.1007/s00705-003-0271-x

[2] Fraser GC, Hooper PT, Lunt RA, Gould AR, Gleeson LJ, et al (1996) Encephalitis caused by a Lyssavirus in fruit bats in Australia. Emerg Infect Dis 2: 327–331. 8969249 10.3201/eid0204.960408 PMC2639915

[3] Harris SL, Mansfield K, Marston DA, Johnson N, Pajamo K, et al (2007) Isolation of European bat lyssavirus type 2 from a Daubenton’s bat (Myotis daubentonii) in Shropshire. Vet Rec 161: 384–386. 17873269 10.1136/vr.161.11.384

[4] Pfefferle S, Oppong S, Drexler JF, Gloza-Rausch F, Ipsen A, et al (2009) Distant relatives of severe acute respiratory syndrome coronavirus and close relatives of human coronavirus 229E in bats, Ghana. Emerg Infect Dis 15: 1377–1384. 10.3201/eid1509.090224 19788804 PMC2819850

[5] Poon LL, Chu DK, Chan KH, Wong OK, Ellis TM, et al (2005) Identification of a novel coronavirus in bats. J Virol 79: 2001–2009. 15681402 10.1128/JVI.79.4.2001-2009.2005 PMC546586

[6] Tang XC, Zhang JX, Zhang SY, Wang P, Fan XH, et al (2006) Prevalence and genetic diversity of coronaviruses in bats from China. J Virol 80: 7481–7490. 16840328 10.1128/JVI.00697-06 PMC1563713

[7] Li W, Shi Z, Yu M, Ren W, Smith C, et al (2005) Bats are natural reservoirs of SARS-like coronaviruses. Science 310: 676–679. 16195424 10.1126/science.1118391

[8] Ge XY, Li JL, Yang XL, Chmura AA, Zhu G, et al (2013) Isolation and characterization of a bat SARS-like coronavirus that uses the ACE2 receptor. Nature 503: 535–538. 10.1038/nature12711 24172901 PMC5389864

[9] Du L, Zhao G, Kou Z, Ma C, Sun S, et al (2013) Identification of a receptor-binding domain in the S protein of the novel human coronavirus Middle East respiratory syndrome coronavirus as an essential target for vaccine development. J Virol 87: 9939–9942. 10.1128/JVI.01048-13 23824801 PMC3754113

[10] Leroy EM, Kumulungui B, Pourrut X, Rouquet P, Hassanin A, et al (2005) Fruit bats as reservoirs of Ebola virus. Nature 438: 575–576. 16319873 10.1038/438575a

[11] Towner JS, Amman BR, Sealy TK, Carroll SA, Comer JA, et al (2009) Isolation of genetically diverse Marburg viruses from Egyptian fruit bats. PLoS Pathog 5: e1000536 10.1371/journal.ppat.1000536 19649327 PMC2713404

[12] Towner JS, Pourrut X, Albarino CG, Nkogue CN, Bird BH, et al (2007) Marburg virus infection detected in a common African bat. PLoS One 2: e764 17712412 10.1371/journal.pone.0000764 PMC1942080

[13] Sotomayor-Bonilla J, Chaves A, Rico-Chavez O, Rostal MK, Ojeda-Flores R, et al (2014) Dengue virus in bats from southeastern Mexico. Am J Trop Med Hyg 91: 129–131. 10.4269/ajtmh.13-0524 24752688 PMC4080551

[14] Thompson NN, Auguste AJ, Travassos da Rosa AP, Carrington CV, Blitvich BJ, et al (2014) Seroepidemiology of Selected Alphaviruses and Flaviviruses in Bats in Trinidad. Zoonoses Public Health. 10.1111/zph.12175 24751420 PMC7165661

[15] Tong S, Li Y, Rivailler P, Conrardy C, Castillo DA, et al (2012) A distinct lineage of influenza A virus from bats. Proc Natl Acad Sci U S A 109: 4269–4274. 10.1073/pnas.1116200109 22371588 PMC3306675

[16] Sun X, Shi Y, Lu X, He J, Gao F, et al (2013) Bat-derived influenza hemagglutinin H17 does not bind canonical avian or human receptors and most likely uses a unique entry mechanism. Cell Rep 3: 769–778. 10.1016/j.celrep.2013.01.025 23434510

[17] Tong S, Zhu X, Li Y, Shi M, Zhang J, et al (2013) New world bats harbor diverse influenza A viruses. PLoS Pathog 9: e1003657 10.1371/journal.ppat.1003657 24130481 PMC3794996

[18] Drexler JF, Corman VM, Muller MA, Maganga GD, Vallo P, et al (2012) Bats host major mammalian paramyxoviruses. Nat Commun 3: 796 10.1038/ncomms1796 22531181 PMC3343228

[19] Kurth A, Kohl C, Brinkmann A, Ebinger A, Harper JA, et al (2012) Novel paramyxoviruses in free-ranging European bats. PLoS One 7: e38688 10.1371/journal.pone.0038688 22737217 PMC3380927

[20] Calisher CH, Childs JE, Field HE, Holmes KV, Schountz T (2006) Bats: important reservoir hosts of emerging viruses. Clin Microbiol Rev 19: 531–545. 16847084 10.1128/CMR.00017-06 PMC1539106

[21] Smith I, Wang LF (2013) Bats and their virome: an important source of emerging viruses capable of infecting humans. Curr Opin Virol 3: 84–91. 10.1016/j.coviro.2012.11.006 23265969 PMC7102720

[22] Taniguchi S, Watanabe S, Masangkay JS, Omatsu T, Ikegami T, et al (2011) Reston Ebolavirus antibodies in bats, the Philippines. Emerg Infect Dis 17: 1559–1560. 10.3201/eid1708.101693 21801651 PMC3381561

[23] Halpin K, Hyatt AD, Fogarty R, Middleton D, Bingham J, et al (2011) Pteropid bats are confirmed as the reservoir hosts of henipaviruses: a comprehensive experimental study of virus transmission. Am J Trop Med Hyg 85: 946–951. 10.4269/ajtmh.2011.10-0567 22049055 PMC3205647

[24] Young PL, Halpin K, Selleck PW, Field H, Gravel JL, et al (1996) Serologic evidence for the presence in Pteropus bats of a paramyxovirus related to equine morbillivirus. Emerg Infect Dis 2: 239–240. 8903239 10.3201/eid0203.960315 PMC2626799

[25] Field H, de Jong C, Melville D, Smith C, Smith I, et al (2011) Hendra virus infection dynamics in Australian fruit bats. PLoS One 6: e28678 10.1371/journal.pone.0028678 22174865 PMC3235146

[26] Smith I, Broos A, de Jong C, Zeddeman A, Smith C, et al (2011) Identifying Hendra virus diversity in pteropid bats. PLoS One 6: e25275 10.1371/journal.pone.0025275 21980413 PMC3182206

[27] Chua KB, Koh CL, Hooi PS, Wee KF, Khong JH, et al (2002) Isolation of Nipah virus from Malaysian Island flying-foxes. Microbes Infect 4: 145–151. 11880045 10.1016/s1286-4579(01)01522-2

[28] Epstein JH, Prakash V, Smith CS, Daszak P, McLaughlin AB, et al (2008) Henipavirus infection in fruit bats (Pteropus giganteus), India. Emerg Infect Dis 14: 1309–1311. 10.3201/eid1408.071492 18680665 PMC2600370

[29] Yadav PD, Raut CG, Shete AM, Mishra AC, Towner JS, et al (2012) Detection of Nipah virus RNA in fruit bat (Pteropus giganteus) from India. Am J Trop Med Hyg 87: 576–578. 10.4269/ajtmh.2012.11-0416 22802440 PMC3435367

[30] Selvey LA, Wells RM, McCormack JG, Ansford AJ, Murray K, et al (1995) Infection of humans and horses by a newly described morbillivirus. Med J Aust 162: 642–645. 7603375 10.5694/j.1326-5377.1995.tb126050.x

[31] Field H, Crameri G, Kung NY, Wang LF (2012) Ecological aspects of hendra virus. Curr Top Microbiol Immunol 359: 11–23. 10.1007/82_2012_214 22476530

[32] Clayton BA, Wang LF, Marsh GA (2013) Henipaviruses: an updated review focusing on the pteropid reservoir and features of transmission. Zoonoses Public Health 60: 69–83. 10.1111/j.1863-2378.2012.01501.x 22709528

[33] Harcourt BH, Tamin A, Ksiazek TG, Rollin PE, Anderson LJ, et al (2000) Molecular characterization of Nipah virus, a newly emergent paramyxovirus. Virology 271: 334–349. 10860887 10.1006/viro.2000.0340

[34] Breed AC, Breed MF, Meers J, Field HE (2011) Evidence of endemic Hendra virus infection in flying-foxes (Pteropus conspicillatus)—implications for disease risk management. PLoS One 6: e28816 10.1371/journal.pone.0028816 22194920 PMC3237542

[35] Mahalingam S, Herrero LJ, Playford EG, Spann K, Herring B, et al (2012) Hendra virus: an emerging paramyxovirus in Australia. Lancet Infect Dis 12: 799–807. 10.1016/S1473-3099(12)70158-5 22921953

[36] Yob JM, Field H, Rashdi AM, Morrissy C, van der Heide B, et al (2001) Nipah virus infection in bats (order Chiroptera) in peninsular Malaysia. Emerg Infect Dis 7: 439–441. 11384522 10.3201/eid0703.010312 PMC2631791

[37] Marsh GA, de Jong C, Barr JA, Tachedjian M, Smith C, et al (2012) Cedar virus: a novel Henipavirus isolated from Australian bats. PLoS Pathog 8: e1002836 10.1371/journal.ppat.1002836 22879820 PMC3410871

[38] Drexler JF, Corman VM, Gloza-Rausch F, Seebens A, Annan A, et al (2009) Henipavirus RNA in African bats. PLoS One 4: e6367 10.1371/journal.pone.0006367 19636378 PMC2712088

[39] Peel AJ, Baker KS, Crameri G, Barr JA, Hayman DT, et al (2012) Henipavirus neutralising antibodies in an isolated island population of African fruit bats. PLoS One 7: e30346 10.1371/journal.pone.0030346 22253928 PMC3257271

[40] Bossart KN, Wang LF, Flora MN, Chua KB, Lam SK, et al (2002) Membrane fusion tropism and heterotypic functional activities of the Nipah virus and Hendra virus envelope glycoproteins. J Virol 76: 11186–11198. 12388678 10.1128/JVI.76.22.11186-11198.2002 PMC136767

[41] Bossart KN, Wang LF, Eaton BT, Broder CC (2001) Functional expression and membrane fusion tropism of the envelope glycoproteins of Hendra virus. Virology 290: 121–135. 11882997 10.1006/viro.2001.1158

[42] Wang L, Harcourt BH, Yu M, Tamin A, Rota PA, et al (2001) Molecular biology of Hendra and Nipah viruses. Microbes Infect 3: 279–287. 11334745 10.1016/s1286-4579(01)01381-8

[43] Bolt G, Pedersen IR (1998) The role of subtilisin-like proprotein convertases for cleavage of the measles virus fusion glycoprotein in different cell types. Virology 252: 387–398. 9878618 10.1006/viro.1998.9464

[44] Garten W, Hallenberger S, Ortmann D, Schafer W, Vey M, et al (1994) Processing of viral glycoproteins by the subtilisin-like endoprotease furin and its inhibition by specific peptidylchloroalkylketones. Biochimie 76: 217–225. 7819326 10.1016/0300-9084(94)90149-x

[45] Gotoh B, Ohnishi Y, Inocencio NM, Esaki E, Nakayama K, et al (1992) Mammalian subtilisin-related proteinases in cleavage activation of the paramyxovirus fusion glycoprotein: superiority of furin/PACE to PC2 or PC1/PC3. J Virol 66: 6391–6397. 1404596 10.1128/jvi.66.11.6391-6397.1992 PMC240131

[46] Smith EC, Popa A, Chang A, Masante C, Dutch RE (2009) Viral entry mechanisms: the increasing diversity of paramyxovirus entry. FEBS J 276: 7217–7227. 10.1111/j.1742-4658.2009.07401.x 19878307 PMC2795005

[47] Pager CT, Dutch RE (2005) Cathepsin L is involved in proteolytic processing of the Hendra virus fusion protein. J Virol 79: 12714–12720. 16188974 10.1128/JVI.79.20.12714-12720.2005 PMC1235853

[48] Pager CT, Craft WW Jr., Patch J, Dutch RE (2006) A mature and fusogenic form of the Nipah virus fusion protein requires proteolytic processing by cathepsin L. Virology 346: 251–257. 16460775 10.1016/j.virol.2006.01.007 PMC7111743

[49] Diederich S, Moll M, Klenk HD, Maisner A (2005) The nipah virus fusion protein is cleaved within the endosomal compartment. J Biol Chem 280: 29899–29903. 15961384 10.1074/jbc.M504598200

[50] Vogt C, Eickmann M, Diederich S, Moll M, Maisner A (2005) Endocytosis of the Nipah virus glycoproteins. J Virol 79: 3865–3872. 15731282 10.1128/JVI.79.6.3865-3872.2005 PMC1075720

[51] Meulendyke KA, Wurth MA, McCann RO, Dutch RE (2005) Endocytosis plays a critical role in proteolytic processing of the Hendra virus fusion protein. J Virol 79: 12643–12649. 16188966 10.1128/JVI.79.20.12643-12649.2005 PMC1235849

[52] Pager CT, Wurth MA, Dutch RE (2004) Subcellular localization and calcium and pH requirements for proteolytic processing of the Hendra virus fusion protein. J Virol 78: 9154–9163. 15308711 10.1128/JVI.78.17.9154-9163.2004 PMC506929

[53] Popa A, Carter JR, Smith SE, Hellman L, Fried MG, et al (2012) Residues in the hendra virus fusion protein transmembrane domain are critical for endocytic recycling. J Virol 86: 3014–3026. 10.1128/JVI.05826-11 22238299 PMC3302302

[54] Diederich S, Sauerhering L, Weis M, Altmeppen H, Schaschke N, et al (2012) Activation of the Nipah virus fusion protein in MDCK cells is mediated by cathepsin B within the endosome-recycling compartment. J Virol 86: 3736–3745. 10.1128/JVI.06628-11 22278224 PMC3302499

[55] Diederich S, Dietzel E, Maisner A (2009) Nipah virus fusion protein: influence of cleavage site mutations on the cleavability by cathepsin L, trypsin and furin. Virus Res 145: 300–306. 10.1016/j.virusres.2009.07.020 19665506 PMC7126315

[56] Michalski WP, Crameri G, Wang L, Shiell BJ, Eaton B (2000) The cleavage activation and sites of glycosylation in the fusion protein of Hendra virus. Virus Res 69: 83–93. 11018278 10.1016/s0168-1702(00)00169-6

[57] Moll M, Diederich S, Klenk HD, Czub M, Maisner A (2004) Ubiquitous activation of the Nipah virus fusion protein does not require a basic amino acid at the cleavage site. J Virol 78: 9705–9712. 15331703 10.1128/JVI.78.18.9705-9712.2004 PMC514977

[58] Craft WW RE Jr. (2005) Sequence motif upstream of the Hendra virus fusion protein cleavage site is not sufficient to promote efficient proteolytic processing. Virology 341: 130–140. 16083935 10.1016/j.virol.2005.07.004

[59] Li Z, Sergel T, Razvi E, Morrison T (1998) Effect of cleavage mutants on syncytium formation directed by the wild-type fusion protein of Newcastle disease virus. J Virol 72: 3789–3795. 9557661 10.1128/jvi.72.5.3789-3795.1998 PMC109601

[60] Paterson RG, Shaughnessy MA, Lamb RA (1989) Analysis of the relationship between cleavability of a paramyxovirus fusion protein and length of the connecting peptide. J Virol 63: 1293–1301. 2644448 10.1128/jvi.63.3.1293-1301.1989 PMC247826

[61] Dubay JW, Dubay SR, Shin HJ, Hunter E (1995) Analysis of the cleavage site of the human immunodeficiency virus type 1 glycoprotein: requirement of precursor cleavage for glycoprotein incorporation. J Virol 69: 4675–4682. 7609032 10.1128/jvi.69.8.4675-4682.1995 PMC189271

[62] Senne DA, Panigrahy B, Kawaoka Y, Pearson JE, Suss J, et al (1996) Survey of the hemagglutinin (HA) cleavage site sequence of H5 and H7 avian influenza viruses: amino acid sequence at the HA cleavage site as a marker of pathogenicity potential. Avian Dis 40: 425–437. 8790895

[63] Simmons G, Gosalia DN, Rennekamp AJ, Reeves JD, Diamond SL, et al (2005) Inhibitors of cathepsin L prevent severe acute respiratory syndrome coronavirus entry. Proc Natl Acad Sci U S A 102: 11876–11881. 16081529 10.1073/pnas.0505577102 PMC1188015

[64] Schornberg K, Matsuyama S, Kabsch K, Delos S, Bouton A, et al (2006) Role of endosomal cathepsins in entry mediated by the Ebola virus glycoprotein. J Virol 80: 4174–4178. 16571833 10.1128/JVI.80.8.4174-4178.2006 PMC1440424

[65] Chandran K, Sullivan NJ, Felbor U, Whelan SP, Cunningham JM (2005) Endosomal proteolysis of the Ebola virus glycoprotein is necessary for infection. Science 308: 1643–1645. 15831716 10.1126/science.1110656 PMC4797943

[66] Pourrut X, Souris M, Towner JS, Rollin PE, Nichol ST, et al (2009) Large serological survey showing cocirculation of Ebola and Marburg viruses in Gabonese bat populations, and a high seroprevalence of both viruses in Rousettus aegyptiacus. BMC Infect Dis 9: 159 10.1186/1471-2334-9-159 19785757 PMC2761397

[67] Crameri G, Todd S, Grimley S, McEachern JA, Marsh GA, et al (2009) Establishment, immortalisation and characterisation of pteropid bat cell lines. PLoS One 4: e8266 10.1371/journal.pone.0008266 20011515 PMC2788226

[68] Jordan I, Horn D, Oehmke S, Leendertz FH, Sandig V (2009) Cell lines from the Egyptian fruit bat are permissive for modified vaccinia Ankara. Virus Res 145: 54–62. 10.1016/j.virusres.2009.06.007 19540275 PMC7172177

[69] Smith EC, Culler MR, Hellman LM, Fried MG, Creamer TP, et al (2012) Beyond anchoring: the expanding role of the hendra virus fusion protein transmembrane domain in protein folding, stability, and function. J Virol 86: 3003–3013. 10.1128/JVI.05762-11 22238302 PMC3302297

[70] Bourne GL, Grainger DJ (2011) Development and characterisation of an assay for furin activity. J Immunol Methods 364: 101–108. 10.1016/j.jim.2010.11.008 21112328

[71] Papenfuss AT, Baker ML, Feng ZP, Tachedjian M, Crameri G, et al (2012) The immune gene repertoire of an important viral reservoir, the Australian black flying fox. BMC Genomics 13: 261 10.1186/1471-2164-13-261 22716473 PMC3436859

[72] Zhang G, Cowled C, Shi Z, Huang Z, Bishop-Lilly KA, et al (2013) Comparative analysis of bat genomes provides insight into the evolution of flight and immunity. Science 339: 456–460. 10.1126/science.1230835 23258410 PMC8782153

[73] Thompson JD, Higgins DG, Gibson TJ (1994) CLUSTAL W: improving the sensitivity of progressive multiple sequence alignment through sequence weighting, position-specific gap penalties and weight matrix choice. Nucleic Acids Res 22: 4673–4680. 7984417 10.1093/nar/22.22.4673 PMC308517

[74] Krahling V, Dolnik O, Kolesnikova L, Schmidt-Chanasit J, Jordan I, et al (2010) Establishment of fruit bat cells (Rousettus aegyptiacus) as a model system for the investigation of filoviral infection. PLoS Negl Trop Dis 4: e802 10.1371/journal.pntd.0000802 20808767 PMC2927428

[75] Wilcox D, Mason RW (1992) Inhibition of cysteine proteinases in lysosomes and whole cells. Biochem J 285 (Pt 2): 495–502. 1637341 10.1042/bj2850495 PMC1132815

[76] Matlin KS, Simons K (1983) Reduced temperature prevents transfer of a membrane glycoprotein to the cell surface but does not prevent terminal glycosylation. Cell 34: 233–243. 6883510 10.1016/0092-8674(83)90154-x

[77] Griffiths G, Pfeiffer S, Simons K, Matlin K (1985) Exit of newly synthesized membrane proteins from the trans cisterna of the Golgi complex to the plasma membrane. J Cell Biol 101: 949–964. 2863275 10.1083/jcb.101.3.949 PMC2113726

[78] Schafer W, Stroh A, Berghofer S, Seiler J, Vey M, et al (1995) Two independent targeting signals in the cytoplasmic domain determine trans-Golgi network localization and endosomal trafficking of the proprotein convertase furin. EMBO J 14: 2424–2435. 7781597 10.1002/j.1460-2075.1995.tb07240.x PMC398356

[79] Thomas G (2002) Furin at the cutting edge: from protein traffic to embryogenesis and disease. Nat Rev Mol Cell Biol 3: 753–766. 12360192 10.1038/nrm934 PMC1964754

[80] Plaimauer B, Mohr G, Wernhart W, Himmelspach M, Dorner F, et al (2001) ‘Shed’ furin: mapping of the cleavage determinants and identification of its C-terminus. Biochem J 354: 689–695. 11237874 10.1042/0264-6021:3540689 PMC1221701

[81] Stieneke-Grober A, Vey M, Angliker H, Shaw E, Thomas G, et al (1992) Influenza virus hemagglutinin with multibasic cleavage site is activated by furin, a subtilisin-like endoprotease. EMBO J 11: 2407–2414. 1628614 10.1002/j.1460-2075.1992.tb05305.x PMC556715

[82] Tian S, Jianhua W (2010) Comparative study of the binding pockets of mammalian proprotein convertases and its implications for the design of specific small molecule inhibitors. Int J Biol Sci 6: 89–95. 20151049 10.7150/ijbs.6.89 PMC2820236

[83] Angliker H, Wikstrom P, Shaw E, Brenner C, Fuller RS (1993) The synthesis of inhibitors for processing proteinases and their action on the Kex2 proteinase of yeast. Biochem J 293 (Pt 1): 75–81. 8328974 10.1042/bj2930075 PMC1134322

[84] Stadler K, Allison SL, Schalich J, Heinz FX (1997) Proteolytic activation of tick-borne encephalitis virus by furin. J Virol 71: 8475–8481. 9343204 10.1128/jvi.71.11.8475-8481.1997 PMC192310

[85] Volchkov VE, Feldmann H, Volchkova VA, Klenk HD (1998) Processing of the Ebola virus glycoprotein by the proprotein convertase furin. Proc Natl Acad Sci U S A 95: 5762–5767. 9576958 10.1073/pnas.95.10.5762 PMC20453

[86] Ortmann D, Ohuchi M, Angliker H, Shaw E, Garten W, et al (1994) Proteolytic cleavage of wild type and mutants of the F protein of human parainfluenza virus type 3 by two subtilisin-like endoproteases, furin and Kex2. J Virol 68: 2772–2776. 8139055

[87] Sugrue RJ, Brown C, Brown G, Aitken J, Mc LRHW (2001) Furin cleavage of the respiratory syncytial virus fusion protein is not a requirement for its transport to the surface of virus-infected cells. J Gen Virol 82: 1375–1386. 11369882 10.1099/0022-1317-82-6-1375

[88] Lindblad-Toh K, Garber M, Zuk O, Lin MF, Parker BJ, et al (2011) A high-resolution map of human evolutionary constraint using 29 mammals. Nature 478: 476–482. 10.1038/nature10530 21993624 PMC3207357

[89] Thompson JD, Gibson TJ, Plewniak F, Jeanmougin F, Higgins DG (1997) The CLUSTAL_X windows interface: flexible strategies for multiple sequence alignment aided by quality analysis tools. Nucleic Acids Res 25: 4876–4882. 9396791 10.1093/nar/25.24.4876 PMC147148

[90] Henrich S, Cameron A, Bourenkov GP, Kiefersauer R, Huber R, et al (2003) The crystal structure of the proprotein processing proteinase furin explains its stringent specificity. Nat Struct Biol 10: 520–526. 12794637 10.1038/nsb941

[91] Bosshart H, Humphrey J, Deignan E, Davidson J, Drazba J, et al (1994) The cytoplasmic domain mediates localization of furin to the trans-Golgi network en route to the endosomal/lysosomal system. J Cell Biol 126: 1157–1172. 7914893 10.1083/jcb.126.5.1157 PMC2120164

[92] Jones BG, Thomas L, Molloy SS, Thulin CD, Fry MD, et al (1995) Intracellular trafficking of furin Is modulated by the phosphorylation state of a casein kinase-Ii site in Its cytoplasmic tail. EMBO Journal 14: 5869–5883. 8846780 10.1002/j.1460-2075.1995.tb00275.x PMC394705

[93] Takahashi S, Nakagawa T, Banno T, Watanabe T, Murakami K, et al (1995) Localization of furin to the trans-Golgi network and recycling from the cell surface involves Ser and Tyr residues within the cytoplasmic domain. J Biol Chem 270: 28397–28401. 7499343 10.1074/jbc.270.47.28397

[94] Wong S, Lau S, Woo P, Yuen KY (2007) Bats as a continuing source of emerging infections in humans. Rev Med Virol 17: 67–91. 17042030 10.1002/rmv.520 PMC7169091

[95] Hoffmann M, Muller MA, Drexler JF, Glende J, Erdt M, et al (2013) Differential sensitivity of bat cells to infection by enveloped RNA viruses: coronaviruses, paramyxoviruses, filoviruses, and influenza viruses. PLoS One 8: e72942 10.1371/journal.pone.0072942 24023659 PMC3758312

[96] Kuhl A, Hoffmann M, Muller MA, Munster VJ, Gnirss K, et al (2011) Comparative analysis of Ebola virus glycoprotein interactions with human and bat cells. J Infect Dis 204 Suppl 3: S840–849. 10.1093/infdis/jir306 21987760 PMC3189982

[97] Kruger N, Hoffmann M, Weis M, Drexler JF, Muller MA, et al (2013) Surface glycoproteins of an African henipavirus induce syncytium formation in a cell line derived from an African fruit bat, Hypsignathus monstrosus. J Virol 87: 13889–13891. 10.1128/JVI.02458-13 24067951 PMC3838219

[98] Weis M, Behner L, Hoffmann M, Kruger N, Herrler G, et al (2014) Characterization of African bat henipavirus GH-M74a glycoproteins. J Gen Virol 95: 539–548. 10.1099/vir.0.060632-0 24296468

[99] Han J, Wang Y, Wang S, Chi C (2008) Interaction of Mint3 with Furin regulates the localization of Furin in the trans-Golgi network. J Cell Sci 121: 2217–2223. 10.1242/jcs.019745 18544638

[100] Hatsuzawa K, Hosaka M, Nakagawa T, Nagase M, Shoda A, et al (1990) Structure and expression of mouse furin, a yeast Kex2-related protease. Lack of processing of coexpressed prorenin in GH4C1 cells. J Biol Chem 265: 22075–22078. 2266110

[101] Schalken JA, Roebroek AJ, Oomen PP, Wagenaar SS, Debruyne FM, et al (1987) fur gene expression as a discriminating marker for small cell and nonsmall cell lung carcinomas. J Clin Invest 80: 1545–1549. 2824565 10.1172/JCI113240 PMC442422

